# The complete chloroplast genome sequence of *Styrax obassia* (Styracaceae)

**DOI:** 10.1080/23802359.2019.1710290

**Published:** 2020-01-14

**Authors:** Xiaogang Xu, Lili Tong, Yaoqin Zhang, Chongli Xia, Yabo Wang

**Affiliations:** a College of Biology and Environment, Nanjing Forestry University, Nanjing, China;; b Co-Innovation Center for Sustainable Forestry in Southern China, Nanjing Forestry University, Nanjing, China;; c School of Horticulture and Landscape Architecture, Jinling Institute of Technology, Nanjing, China

**Keywords:** *Styrax obassia*, Styracaceae, complete chloroplast genome, phylogenomics

## Abstract

*Styrax obassia* Siebold & Zucc. is a distinct member of the family Styracaceae with fragrant and beautiful flowers. In this study, we determined the complete chloroplast (cp) genome sequence of *S. obassia* in an effort to provide genomic resources useful for promoting its conservation. The circular genome of *S. obassia* was 157,910 bp in size and contained two inverted repeat (IRa and IRb) regions of 26,051 bp, which were separated by a large single copy (LSC) region of 87,528 bp, and a small single copy (SSC) region of 18,280 bp. A total of 133 genes are encoded, including 88 protein-coding genes, 37 tRNA genes, and eight rRNA genes. The overall GC content of *S. obassia* genome is 36.97%. The phylogenetic analysis suggests that *S. obassia* is a sister species to *Styrax suberifolius* and *Styrax zhejiangensis* in Styracaceae.

*Styrax obassia* Siebold & Zucc., is a broad-leaved tree species with fragrant and beautiful flowers, which is mainly distributed northern China which has high medicinal value and can be as applied for ornamental purposes. Despite this progress in understanding Styracaceae systematics, many of the relationships among species or genera in the family remain poorly resolved. Comparison of complete plastome sequences further provides the opportunity to explore sequence variation and molecular evolutionary patterns associated with gene loss, rearrangements, duplication, and transfer events. Here, we characterized the complete cp genome sequence of *S. obassia* (GeneBank accession number: MN560143) based on Illumina pair-end sequencing to provide a valuable complete cp genomic resource.

The total genomic DNA was extracted from the fresh leaves of *S. obassia* grown in Zhongshan Botanical Garden (N 32.1566, E 118.9690) in Nanjing, Jiangsu, China. The voucher specimen was kept in the herbarium of Nanjing Forestry University (accession number: NF2019061). The whole genome sequencing was conducted by Nanjing Genepioneer Biotechnologies Inc. (Nanjing, China) on the Illumina Hiseq platform. The raw reads were filtered by CLC Genomics Workbench v9, and the obtained clean reads were assembled into chloroplast genome using SPAdes assembler v3.10.1 (Bankevich et al. 2012). Finally, gene structure annotation was carried out with CpGAVAS (Liu et al. 2012), and equences were aligned by MAFFT v7.307 (Katoh and Standley 2014). A Neighbor-Joining (NJ) tree with 100 bootstrap replicates was inferred using MEGA version 7 (Kumar et al. 2016).

The circular genome of *S. obassia* was 157,910 bp in size and contained two inverted repeat (IRa and IRb) regions of 26,051 bp, which were separated by a large single copy (LSC) region of 87,528 bp, and a small single copy (SSC) region of 18,280 bp. A total of 133 genes are encoded, including 88 protein-coding genes (81 PCG species), 37 tRNA genes (30 tRNA species), and eight rRNA genes (four rRNA species). Most of genes occurred in a single copy, however, seven protein-coding genes (*ndhB, rpl2, rpl23, rps12, rps7, ycf15,* and *ycf2*), seven tRNA genes (*trnA-UGC, trnI-CAU, trnI-GAU, trnL-CAA, trnN-GUU, trnR-ACG,* and *trnV-GAC)* , and four rRNA genes (*16S, 23S, 4.5S,* and *5S*) are totally duplicated. A total of nine protein-coding genes (*atpF, ndhA, ndhB, petB, petD, rpl16, rpl2, rpoC1,* and *rps16*) contained one intron while the other three genes (*clpP, rps12, and ycf3*) had two intron each. The overall GC content of *S. obassia* genome is 36.97%, and the corresponding values in LSC, SSC and IR regions are 34.81%, 30.31% and 42.92%, respectively.

To ascertain the phylogenetic evolution of *S. obassia*, the fasta format file containing all the chloroplast genome sequences of 33 species (28 Styracaceae chloroplast genomes, 2 Symplocaceae chloroplast genomes, 1 Clethraceae chloroplast genomes, and 2 Ericaceae chloroplast genomestaxa). The phylogenetic analysis suggests that *S. obassia* is a sister species to *Styrax suberifolius* and *Styrax zhejiangensis* in Styracaceae, with bootstrap support values of 100% (Figure 1).

**Figure 1.**
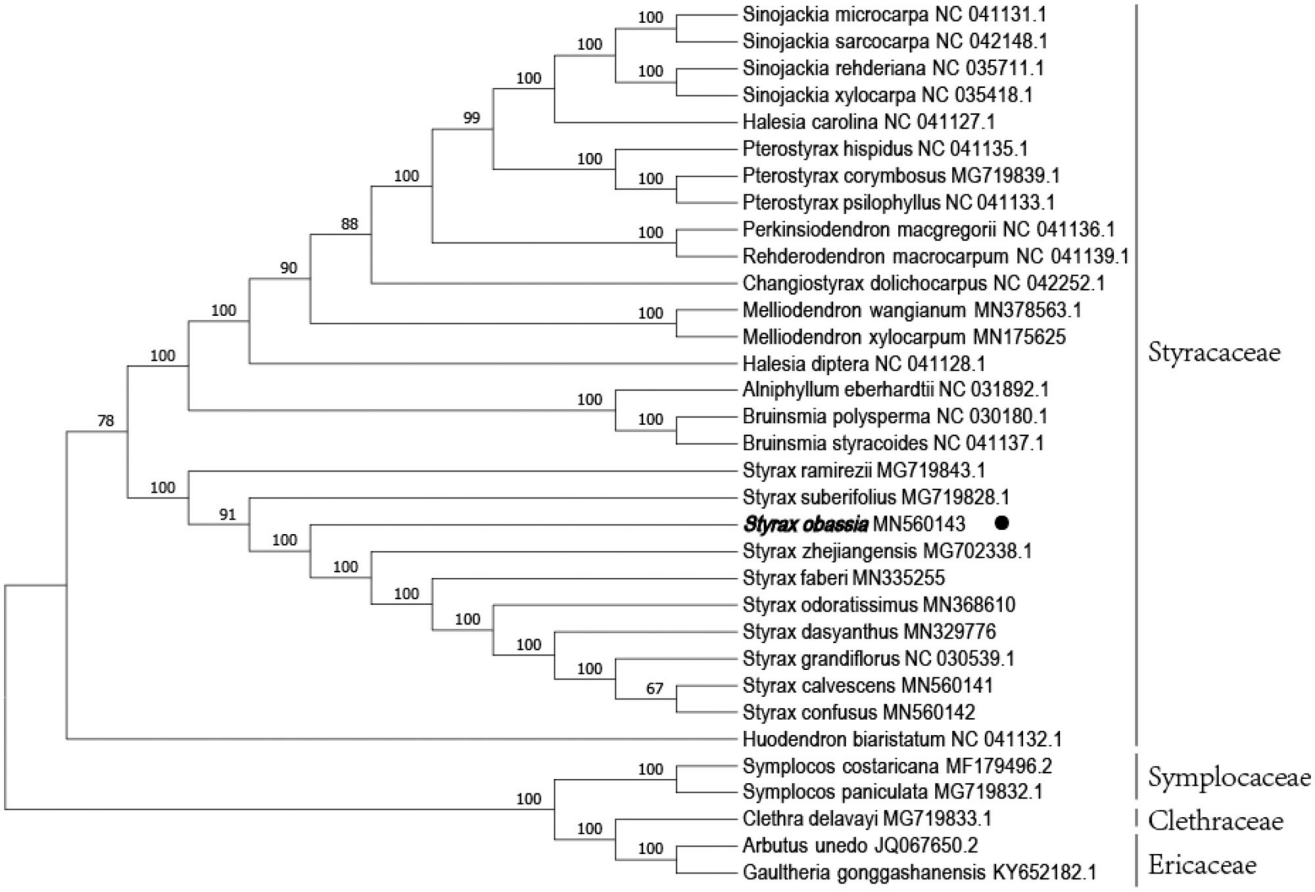
Phylogenetic tree inferred by Neighbor-Joining (NJ) method based on the complete chloroplast genome of 33 representative species. The bootstrap support values are shown at the branches.
